# Position-correlated biphoton wavefront sensing for quantum adaptive imaging

**DOI:** 10.1038/s41377-025-02024-4

**Published:** 2025-09-08

**Authors:** Yi Zheng, Zhao-Di Liu, Jian-Shun Tang, Jin-Shi Xu, Chuan-Feng Li, Guang-Can Guo

**Affiliations:** 1 Laboratory of Quantum Information, University of Science and Technology of China, 230026 Hefei, China; 2 Anhui Province Key Laboratory of Quantum Network, University of Science and Technology of China, 230026 Hefei, China; 3 CAS Center for Excellence in Quantum Information and Quantum Physics, University of Science and Technology of China, 230026 Hefei, China; 4 Hefei National Laboratory, University of Science and Technology of China, 230088 Hefei, China

**Keywords:** Quantum optics, Imaging and sensing, Adaptive optics

## Abstract

Quantum imaging with spatially entangled photons offers advantages such as enhanced spatial resolution, robustness against noise, and counterintuitive phenomena, while a biphoton spatial aberration generally degrades its performance. Biphoton aberration correction has been achieved by using classical beams to detect the aberration source or scanning the correction phase on biphotons if the source is unreachable. Here, a new method named position-correlated biphoton Shack–Hartmann wavefront sensing is introduced, where the phase pattern added on photon pairs with a strong position correlation is reconstructed from their position centroid distribution at the back focal plane of a microlens array. Experimentally, biphoton phase measurement and adaptive imaging against the disturbance of a plastic film are demonstrated. This single-shot method is a more direct and efficient approach toward quantum adaptive optics, suitable for integration into quantum microscopy, remote imaging, and communication.

## Abstract (web summary)

Inspired by classical Shack–Hartmann wavefront sensing, the spatial aberration of position-correlated biphotons can be efficiently measured using a microlens array, paving the way for genuine quantum adaptive optics.

## Graphical abstract

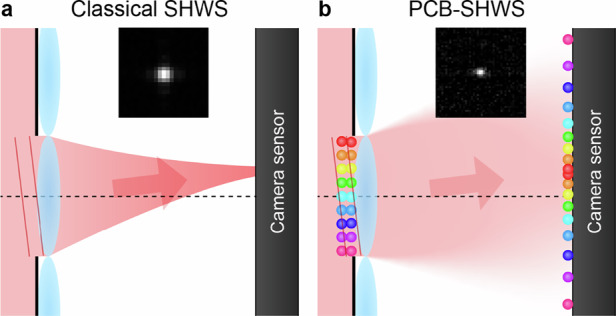

## Introduction

Entangled photons play a critical role in the development of quantum information and technology^1^. Quantum imaging, which fully utilizes the spatial degree of freedom of photons, can achieve several nonclassical optical effects^2,3^. Ghost imaging^4^ and quantum imaging with undetected photons^5^ are counterintuitive imaging methods. By joint probability distribution (JPD) measurement, spatially entangled photons can achieve a higher spatial resolution than the Rayleigh limit^6–8^, which is beneficial in optical microscopy. Also, they can be distilled from the stray light^9,10^. However, the atmospheric turbulence or flaws in optical instruments introduce phase aberrations and degrade the imaging performance in both classical and quantum imaging^11^. Adaptive optics, including phase measurement and correction, is dedicated to overcoming this problem.

In classical optics, a famous phase measurement method is Shack–Hartmann wavefront sensing (SHWS)^12–14^, which uses a microlens array to focus the light inside each aperture. The local obliquity of light within an aperture, corresponding to the phase gradient, is mapped to the displacement of the spot at the microlens back focal plane. The measured gradient distribution is discretized according to the microlens width, which limits its spatial resolution. Then, the phase distribution is reconstructed by the zonal or modal method^15^ and corrected by a spatial light modulator (SLM) or a deformable mirror. There are also other types of wavefront sensors^14,16,17^ and even sensorless adaptive optical techniques. The basic idea of a notable sensorless one is that with an aberration, the focused spot after a Fourier lens increases in size and decreases in peak intensity, which serves as the criterion to perform feedback control of the correction phase till the sharp peak revives^18^.

In quantum optics, the spatial phase of entangled photon pairs (biphotons) can be measured by holography using a reference beam^19,20^ or the polarization entanglement^21,22^, and the aberration cancellation of biphotons correlated in position by measuring the aberration source with classical lights has been demonstrated^23–25^ (in our article, the word “correlated” means the two photons are approximately at the same position, contrary to the term “anti-correlated,” and does not mean they can have an arbitrary nonseparable JPD). On the other hand, if a phase aberration from an unreachable source has already been added to position-correlated biphotons, a prominent measurement method has been demonstrated by Cameron et al.^11^. In their protocol, inspired by the sensorless method^18^, the criterion is the peak value of the biphoton position sum-coordinate (or centroid, equivalently) marginal distribution at the Fourier plane. By scanning the coefficients of Zernike polynomials, the aberration can be eliminated. This indirect way requires multiple measurement steps, so the optimal group of coefficients may not be easily obtained by sequential scanning rather than using special algorithms. In this work, we introduce a single-shot method to realize this task, namely position-correlated biphoton SHWS (PCB-SHWS), which directly measures the gradient of phase added on position-correlated biphotons. Then, using biphotons from spontaneous parametric down-conversion (SPDC)^26,27^, the phase measurement and adaptive imaging is experimentally demonstrated.

## Results

### Theoretical framework

The most fundamental idea of PCB-SHWS is from the Einstein–Podolsky–Rosen paper^28^. Denoting the transverse position ${\boldsymbol{\rho }}=(x,y)$ and momentum ${\bf{q}}=({k}_{x},{k}_{y})$, letting the biphoton field in front of a lens with the focal length ${f}_{{\rm{SH}}}$ have a constant intensity, a perfect position correlation, and the phase of an oblique plane wave ${e}^{i{{\bf{q}}}_{0}\cdot {\boldsymbol{\rho }}}$ added on each photon, the wavefunction (joint amplitude)^20,29,30^ in the position space $\psi ({{\boldsymbol{\rho }}}_{1},{{\boldsymbol{\rho }}}_{2})=\delta ({{\boldsymbol{\rho }}}_{1}-{{\boldsymbol{\rho }}}_{2}){e}^{2i{{\bf{q}}}_{0}\cdot {{\boldsymbol{\rho }}}_{1}}$, and their momenta are perfectly anti-correlated $\tilde{\psi }\left({{\bf{q}}}_{1},{{\bf{q}}}_{2}\right)=\delta ({{\bf{q}}}_{1}+{{\bf{q}}}_{2}-2{{\bf{q}}}_{0})$ with the anti-correlation center ${{\bf{q}}}_{0}$. Denoting their wavelength $\lambda$ and wave number $k=2\pi /\lambda$, the position wavefunction at the back focal plane of the lens is $\tilde{\psi }\left({f}_{{\rm{SH}}}{{\bf{q}}}_{1}/k,{f}_{{\rm{SH}}}{{\bf{q}}}_{2}/k\right)$ with a paraboloid phase added [because $\psi ({{\boldsymbol{\rho }}}_{1},{{\boldsymbol{\rho }}}_{2})$ is not the wavefunction at the front focal plane] which does not affect the JPD ${\Gamma \left({{\bf{q}}}_{1},{{\bf{q}}}_{2}\right)=\left|\widetilde{\psi }\left({{\bf{q}}}_{1},{{\bf{q}}}_{2}\right)\right|}^{2}$ (for simplicity, we directly use ${\bf{q}}$ and ignore the normalization). So, after measuring the JPD, denoting the centroid ${{\bf{q}}}_{c}=({{\bf{q}}}_{1}+{{\bf{q}}}_{2})/2$, summing JPD values of point pairs with the same centroid yields the biphoton centroid marginal distribution

1 $${\Gamma }_{c}({{\bf{q}}}_{c})=\int d{{\bf{q}}}_{1}\Gamma ({{\bf{q}}}_{1},2{{\bf{q}}}_{c}-{{\bf{q}}}_{1})\propto \delta ({{\bf{q}}}_{c}-{{\bf{q}}}_{0})$$

which is a sharp peak at ${{\bf{q}}}_{0}$. When photons at the whole microlens array arrive at its back focal plane, ignoring the momentum anti-correlation weakening caused by the finite microlens width $2a$, if the phase at each aperture can be approximated by an oblique plane wave and the dynamic range of SHWS $|{q}_{0x}|\,,\,|{q}_{0y}|\, < ka/{f}_{{\rm{SH}}}$ is satisfied, photon pairs from different apertures have distinct centroids which are inside their own aperture, and the whole centroid distribution is an array of sharp peaks, similar as the measured intensity distribution of classical SHWS. The gradient values of the whole phase pattern can be calculated from the peak positions relative to their centers.

Then, we consider the effects of the finite microlens width, a finite position correlation with the form $c({{\boldsymbol{\rho }}}_{1}-{{\boldsymbol{\rho }}}_{2})$ whose width is far less than $2a$, and the added phase $\Phi ({\boldsymbol{\rho }})$, which is generally not a plane wave. The position wavefunction at an aperture $S$ centered by ${\bf{0}}$ is

2 $$\psi ({{\boldsymbol{\rho }}}_{1},{{\boldsymbol{\rho }}}_{2})=c({{\boldsymbol{\rho }}}_{1}-{{\boldsymbol{\rho }}}_{2})U({{\boldsymbol{\rho }}}_{1})U({{\boldsymbol{\rho }}}_{2})$$

where $U({\boldsymbol{\rho }})={e}^{i\Phi ({\boldsymbol{\rho }})}\text{rect}[{\boldsymbol{\rho }}/(2a)]$ and the two-dimensional rectangular function $\text{rect}({\boldsymbol{\rho }})=1$ when $|x|\,\le 1/2,\,|y|\,\le 1/2$ and 0 elsewhere. Denoting the Fourier transforms $\tilde{U}\left({\bf{q}}\right)=\int d{\boldsymbol{\rho }}U({\boldsymbol{\rho }}){e}^{-i{\bf{q}}\cdot {\boldsymbol{\rho }}}$ and $\tilde{c}\left({\bf{q}}\right)=\int d{\boldsymbol{\rho }}c({\boldsymbol{\rho }}){e}^{-i{\bf{q}}\cdot {\boldsymbol{\rho }}}$, from the convolution theorem, the momentum wavefunction

3 $$\tilde{\psi }\left({{\bf{q}}}_{1},{{\bf{q}}}_{2}\right)=\left[\tilde{U}\left({{\bf{q}}}_{1}\right)\tilde{U}({{\bf{q}}}_{2})\right]* \left[\tilde{c}\left(\frac{{{\bf{q}}}_{1}-{{\bf{q}}}_{2}}{2}\right)\delta \left({{\bf{q}}}_{1}+{{\bf{q}}}_{2}\right)\right]$$

where “$\ast$” is the convolution sign. In classical SHWS, ${\left|\widetilde{U}\left({\bf{q}}\right)\right|}^{2}$ is the measured intensity from the field $U({\boldsymbol{\rho }})$, and the average ${\bf{q}}$ is the average phase gradient in this aperture^13^

4 $$\langle {\bf{q}}\rangle =\frac{1}{{(2a)}^{2}}{\int }_{S}d{\boldsymbol{\rho }}\nabla \varPhi ({\boldsymbol{\rho }})={\langle \nabla \varPhi ({\boldsymbol{\rho }})\rangle }_{S}$$

For $\Phi(\rho)$ whose gradient is slow-varying within the aperture (in order to satisfy the spatial resolution limit), $\tilde{U}\left({\bf{q}}\right)$ is still peaked at $\langle {\bf{q}}\rangle$, although the exact distribution is a bit different from the sinc function in the plane-wave case. From Eq. (3), considering the displacement and broadening effects of convolution, their momenta are approximately anti-correlated with the center $\langle {\bf{q}}\rangle$, so the measured centroid distribution is peaked at $\langle {\bf{q}}\rangle$. The spot in the direct image is a slightly blurred ${\left|\tilde{c}\left({\bf{q}}\right)\right|}^{2}$, which may be larger than an aperture (see Supplementary Information 2 for the experimental images), as the biphoton field is spatially incoherent in the first order. So, the phase added to such a biphoton field cannot be measured by classical SHWS.

**Fig. 1:**
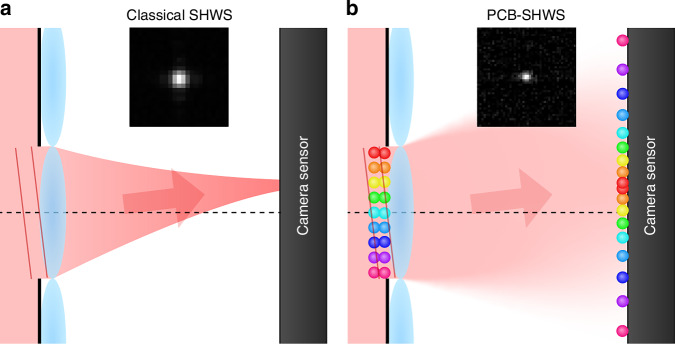
**Principle of classical SHWS and PCB-SHWS.** For simplicity, we show the light fields from a single aperture. **a** Classical SHWS. The inset shows a focused spot inside a microlens aperture with a width 300 μm using an 808-nm laser as the light source, taken by the EMCCD with the EM gain set to 0. **b** PCB-SHWS. At the microlens and its back focal plane, respectively, two balls with the same color represent an entangled photon pair. A tilt phase leads to the centroid displacement. The inset is the biphoton centroid marginal distribution inside an aperture from our phase measurement experiment

Under the approximation of a perfect position correlation $c({\boldsymbol{\rho }})\approx \delta ({\boldsymbol{\rho }})$, Eq. (3) can be further simplified to

5 $$\begin{array}{c}\tilde{\psi }\left({{\bf{q}}}_{1},{{\bf{q}}}_{2}\right)\approx \left[\tilde{U}\left({{\bf{q}}}_{1}\right)\tilde{U}\left({{\bf{q}}}_{2}\right)\right]* \delta \left({{\bf{q}}}_{1}+{{\bf{q}}}_{2}\right)\\ =\int d{\bf{q}}\tilde{U}\left({\bf{q}}\right)\tilde{U}\left({{\bf{q}}}_{1}+{{\bf{q}}}_{2}-{\bf{q}}\right)={\tilde{U}}_{2}\left({{\bf{q}}}_{1}+{{\bf{q}}}_{2}\right)\end{array}$$

where ${\tilde{U}}_{2}\left({\bf{q}}\right)$ is the Fourier transform of ${[U({\boldsymbol{\rho }})]}^{2}$ whose phase is $2\varPhi ({\boldsymbol{\rho }})$. The centroid distribution ${\Gamma }_{c}({{\bf{q}}}_{c})={\left|{\tilde{U}}_{2}\left(2{{\bf{q}}}_{c}\right)\right|}^{2}$, so

6 $$\langle {{\bf{q}}}_{c}\rangle =\frac{1}{2}\frac{1}{{(2a)}^{2}}{\int }_{S}d{\boldsymbol{\rho }}\nabla [2\Phi ({\boldsymbol{\rho }})]={\langle \nabla \Phi ({\boldsymbol{\rho }})\rangle }_{S}$$

exactly corresponds to the average phase gradient in classical SHWS, while the peak width is half the classical result. Experimentally, the centroid distribution can be efficiently measured^31^ and has a twice-pixel resolution as direct images^32^. In spite of these differences, the data processing methods of classical SHWS can be applied to the centroid distribution for phase reconstruction. The principle of classical SHWS and PCB-SHWS is shown in Fig. 1, where the insets are a direct image of classical SHWS and a centroid distribution of PCB-SHWS inside an aperture.

### Experimental setup

**Fig. 2:**
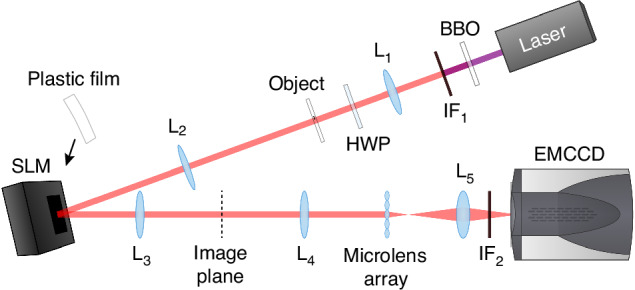
**Experimental setup of PCB-SHWS.** The laser passes through a beam shaping system (not shown) and pumps the BBO crystal. Degenerate collinear type-I down-converted photons from the BBO pass through the first Fourier lens L_1_, the half-wave plate (HWP), the object, and the second Fourier lens L_2_, and are reflected by the SLM. A plastic film may be pasted in front of the SLM. Then, they pass through a 4*f* system L_3_,L_4_ and is incident on the microlens array. An imaging lens L_5_ images the optical field at the microlens back focal plane to the EMCCD sensor. IF_1_: long-pass interference filter; IF_2_: bandpass filter. In the imaging experiment, the EMCCD sensor is moved to the image plane together with IF_2_ (see Supplementary Information 3 for the setup)

We use an electron-multiplying charge-coupled device (EMCCD) camera and the multiple frame method developed by Defienne et al.^33–35^. to measure the biphoton JPD^8,9,11,21–23,30,32,36,37^. The setup of the phase measurement experiment is shown in Fig. 2. A horizontally-polarized (H) laser beam at 404 nm pumps a β-barium borate (BBO) crystal with the thickness 1 mm and is removed by a long-pass interference filter (IF). Degenerate collinear type-I down-converted photon pairs at the vertical (V) polarization pass through the first Fourier lens (${f}_{1}=15\,{\rm{cm}}$) and are switched to H polarization by a half-wave plate. Their positions are anti-correlated at their focal plane, where the object (USAF 1951 resolution target) is placed at one-half of the beam. After the second Fourier lens (${f}_{2}=25\,{\rm{cm}}$), an SLM adds the same phase pattern to each photon. Then, they pass through a 4 *f* system (${f}_{3}={f}_{4}=15\,{\rm{cm}}$) and arrive at the microlens array with the aperture width 300 μm and focal length ${f}_{{\rm{SH}}}=14.6\,{\rm{mm}}$. An imaging lens (${f}_{5}=5\,{\rm{cm}}$) projects the optical field at the microlens back focal plane to the EMCCD sensor with the magnification ratio −1, and a bandpass IF selects near-degenerate down-converted photons. See Materials and methods for details.

### Biphoton phase measurement

As the region of interest (ROI) in our experiment is a square, we use the modal method based on two-dimensional Legendre polynomials^38^ ${L}_{m,n}({\boldsymbol{\rho }})={L}_{m}(x){L}_{n}(y)$ to reconstruct the phase distribution from the gradient, rather than Zernike polynomials which are suitable for circular ROIs. See Materials and methods and Supplementary Information 1 for data processing algorithms.

**Fig. 3:**
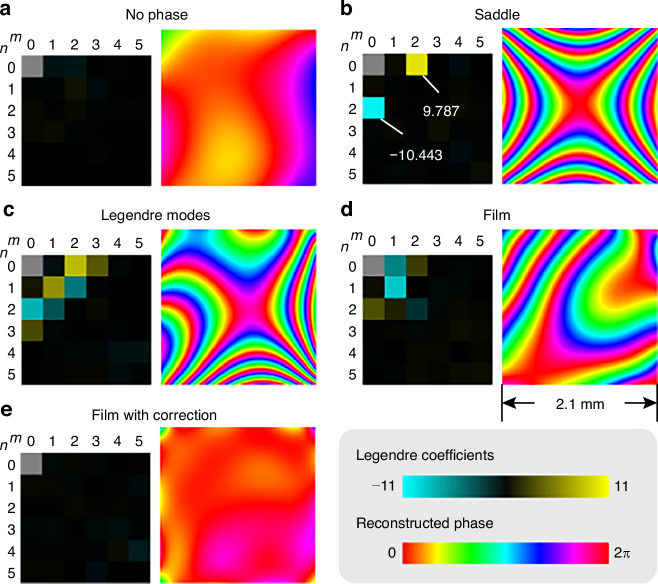
Biphoton phase measurement result. The calculated Legendre coefficients (excluding *L*_0,0_, on the left of each panel) and phase distributions (on the right of each panel) are shown for the five cases: **a** no phase, **b** a saddle phase, **c** Legendre modes, **d** a plastic film, and **e** the film with correction. The gradient distribution of the no-phase case is the reference of the other cases

We measure the phase of five cases: no phase added, a hyperbolic paraboloid (saddle) phase $10({L}_{2,0}-{L}_{0,2})$, a superposition of several Legendre modes

7 $$8{L}_{2,0}+6{L}_{1,1}-7{L}_{0,2}+4{L}_{3,0}-5{L}_{2,1}-4{L}_{1,2}+3{L}_{0,3}$$

a plastic film placed in front of the SLM, and the plastic film with phase correction. The exposure times, direct images, centroid distributions, and gradient distributions are shown in Supplementary Information 2. From the direct images, the object pattern is faintly visible in each aperture because it modifies $\tilde{c}\left({\bf{q}}\right)$. The measured gradient distribution of the no-phase case still deviates from zero, which is mainly due to the aberration of the imaging lens, so it serves as a reference by which the measured gradient distributions of other cases are subtracted. The calculated Legendre coefficients (see the tables in Supplementary Information for values) and the reconstructed phase distributions are shown in Fig. 3. In the saddle phase case, the calculated ${L}_{2,0}$ and ${L}_{0,2}$ are close to the theoretical values 10 and −10, respectively. In the cases of Legendre modes and film with correction, ignoring the tilt terms ${L}_{1,0}$ and ${L}_{0,1}$, which do not blur the image, compared to Eq. (7) and 0, respectively, the root mean square error values of the phase distributions (discretized into 120 × 120 pixels; in the unit of optical path difference) are $0.0623\lambda$ and $0.0502\lambda$. So, the measured phases fit well with the designed ones on the SLM, and the aberration caused by the film is approximately corrected. The errors are mainly from the imperfection of SLM, slight misalignment of the setup, image distortion from the imaging lens, and noise from the multiple frame method.

### Adaptive imaging

To test whether the biphoton phase is correctly canceled after PCB-SHWS, we then perform the adaptive imaging experiment. The EMCCD sensor is moved to the back focal plane of the third Fourier lens together with the bandpass IF. See Supplementary Information 3 for a figure of this new setup and a brief principle of biphoton imaging with aberrations. When taking frames, 2 × 2 pixels are binned into one on the EMCCD to increase the frame rate^8^.

**Fig. 4:**
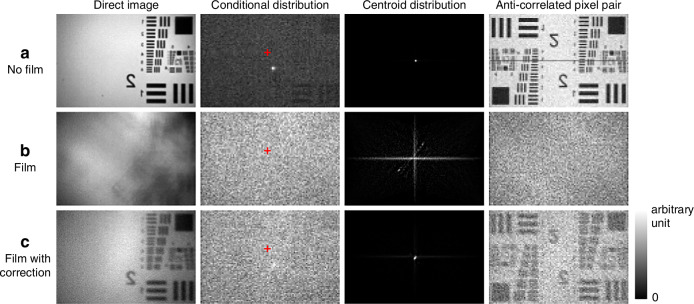
**Adaptive imaging result.** The direct images (without pixel binning, normalized according to the maximum and minimum value), CPDs with the other photon postselected to a certain pixel (the red cross), centroid marginal distributions without background removal (the horizontal and vertical bright lines are noises from the summation which are present in all three cases, but in the no-film and film with correction case, they are less significant because the peaks are much brighter), and joint probabilities of anti-correlated pixel pairs are shown for the **a** no-film, **b** film, and **c** film with correction case. The image sizes are 2.678 × 1.794 mm

We take $4.41\times {10}^{6}$ frames in each of the three cases: no film, film, and film with correction, as well as direct images without pixel binning. The direct images, conditional probability distributions (CPDs) of one photon with the other photon postselected to a certain pixel, centroid distributions, and JPDs of anti-correlated pixel pairs are shown in Fig. 4. The anti-correlated pair distributions have central symmetry, so the object patterns exist on the other halves of the images. When the film is present, the object cannot be identified in the direct image or the anti-correlated pair distribution. After correction, they are visible again, but a bit blurry because the slightly curved film about 2 mm away from the SLM liquid crystal plate cannot be regarded as a pure phase object at exactly the SLM plane. A part of the photons are reflected or scattered after passing through the film twice, equivalent to a lower quantum efficiency of the sensor, so the signal-to-noise ratios (SNRs) of the conditional distribution and the anti-correlated pair distribution in the film with correction case are much lower than the no-film case. The centroid distribution, which is the metric of the sensorless adaptive imaging method^11^, is peaked at the center point in the no-film or film with correction case, while scattered in the film case.

## Discussion

In this work, we introduced PCB-SHWS to measure the phase pattern added on biphotons with a strong position correlation, and performed experiments of Legendre coefficient measurement and adaptive imaging against the disturbance of a plastic film. Both PCB-SHWS and the sensorless approach^11^ do not require classical detection of the aberration source^24,25^, reference beams^19^, or polarization entanglement^21,22^, while our method only requires one measurement step and has the advantage in efficiency. Compared to the multiple frame method, with a more advanced biphoton JPD measurement technique like the time-stamping camera^20,39^, the phase can be rapidly acquired for real-time (truly adaptive) correction of a time-varying aberration, suitable for future quantum communication and imaging research. However, limited by the microlens width and camera pixel width which affect the spatial resolution and sensitivity, respectively, like classical SHWS, the result of PCB-SHWS may not be precise enough. So, in order to restore a quantum image from common aberrations perfectly in quantum microscopy, PCB-SHWS can first provide a good estimate of the phase, and then mode coefficient scanning can precisely determine it. By reducing the microlens size or magnifying the beam, the spatial resolution can be improved to some extent (with the sensitivity reduced), but it is, after all, not applicable to extremely detailed phase patterns such as those from diffusers^23–25^.

If the photons are anti-correlated in position, the effective phase is an even function^35^, and its measurement scheme can also be analyzed, but the lateral position of the microlens array should be adjusted so that the anti-correlation center is the center, a side midpoint, or a vertex of an aperture, as detailed in Supplementary Information 4.

Although the essential sensing setup (a microlens array and a camera capable of JPD measurement) in PCB-SHWS also appears in the method named quantum SHWS from our previous work^30^, their theoretical frameworks, data processing methods, and scopes of application are distinct, and a biphoton state suitable for one method is unsuitable for the other. A clarification of their differences is given in Supplementary Information 5. Also, in classical optics, apart from using only the peak positions for SHWS, the whole momentum distribution from each microlens aperture provides more information about the optical field^40,41^, which has been incorporated in a new technique, namely plenoptic imaging or light-field imaging^42,43^. Therefore, the microlens array, which enables position and local momentum measurement without breaking the uncertainty principle, has great potential in detecting higher-order correlation properties of multiphoton optical fields when combined with efficient photon coincidence detection techniques. Its further applications in quantum optics can be explored in the future.

## Materials and methods

### Details of the experimental setup

Before pumping the BBO, the continuous-wave laser beam (TOPTICA) passes through two Fourier lenses for magnification, two cylindrical lenses to adjust its shape, and a short-pass filter at 600 nm. The long-pass IF is at 647 nm. The SLM (Hamamatsu X13267) has 792 × 600 pixels whose widths are 12.5 μm. We choose ${f}_{2} > {f}_{3}$ because the beam at the back focal plane of the first Fourier lens is too large for the EMCCD (Andor iXon Ultra 888; pixel width: 13 μm) in the imaging setup, and thus the image of the object is shrunk (we focus on proof-of-principle aberration cancellation in this work and do not discuss quantum superresolution imaging or image distillation). The imaging lens is used because the distance between the EMCCD casing and its sensor is 1.75 cm, which is larger than ${f}_{{\rm{SH}}}$, and it is not easy to put the microlens array (LBTEK) inside the casing (a shutter is at its inner side). This will introduce distortions when imaging, so the beam should be at the center of the imaging lens and not too large. The center wavelength of the bandpass IF [(810 ± 5) nm] is not 808 nm, but degenerate photon pairs can pass through it, and the only shortcoming is the existence of some single-photon incidences, which are treated as dark counts.

Using the formulae in ref. ^44^, the full width at half maximum (FWHM) of the biphoton CPD (the two-dimensional probability distribution of one photon when the other is postselected to a given position) is about 17 μm at the BBO and 28 μm at the microlens array, so most of the photon pairs pass through the same microlens; the FWHM of the spot at the back focal plane from one aperture is about 604 μm, larger than the aperture.

The ROI of the EMCCD is set to 165 × 165 pixels in the phase measurement experiment and 105 × 71 (binned) in the imaging one. The EM gain (set to 0 when taking the direct image in Fig. 1a), horizontal pixel readout rate, vertical pixel shift speed, and vertical clock voltage amplitude are set to 1000, 10 MHz, 0.6 μs and +2 V, respectively. The exposure times are different in each measurement, determined by the beam intensity, so that roughly $1/5$ of the pixels in the ROI have photons in each frame^34^ (see Supplementary Information 2 for the values in the phase measurement experiment. In the imaging experiment, the exposure times are 1.4 ms, 1.8 ms, and 2.4 ms for the no-film, film, and film with correction case, respectively. The EMCCD sensor is cooled to −53 °C and −38 °C in the phase measurement and imaging (a higher frame rate produces more heat) experiment, respectively. When taking the three direct images, the exposure time is 0.1 s, and 1000 frames are directly summed for each case.

### Data processing

In the multiple frame method, the EMCCD captures $N$ frames with grayscale values from 0 to 65535, and a threshold binarizes the frame data. In our experiment, by evaluating the grayscale value histogram without the down-converted photons, the threshold is set to 509. Denoting the counting of the *i*th pixel (the indexing is arbitrary) of the *n*th frame as ${C}_{n,i}$ (${C}_{n,i}=0$ or $1$), defining the single-pixel count average $\langle {C}_{i}\rangle =\frac{1}{N}{\sum }_{n=1}^{N}{C}_{n,i}$ and the two-pixel coincidence average $\langle {C}_{ij}\rangle =\frac{1}{N}{\sum }_{n=1}^{N}{C}_{n,i}{C}_{n,j}$, if the Poissonian statistics of photons is assumed, the JPD is estimated by the covariance of the counts of two pixels ${\Gamma }_{ij}\approx \langle {C}_{ij}\rangle -\langle {C}_{i}\rangle \langle {C}_{j}\rangle$. Its derivation has taken the quantum efficiency and the dark count into account^34^. The SNR is a major issue, so it is only suitable for biphoton states with narrow CPDs^36^. The JPD of the same pixel cannot be measured by this method, and JPD of two pixels near each other on the same line has abnormal correlations due to the charge smearing effect of EMCCD^7^, so linear interpolation is applied when necessary: for two pixels on the same line $({x}_{1},y)$ and $({x}_{2},y)$, if $|{x}_{1}-{x}_{2}|\,\le 10$ pixels (no such condition in the imaging experiment), their JPD value ${\Gamma }_{({x}_{1},y),({x}_{2},y)}$ are replaced by $({\Gamma }_{({x}_{1},y),({x}_{2},y+1)}+{\Gamma }_{({x}_{1},y),({x}_{2},y-1)})/2$. If the photons have a strong position anti-correlation, the interpolated result will be lower than normal, as shown in the no-film case in Fig. 4a.

The actual count rate drifts over time, adding a positive background to the calculated ${\Gamma }_{ij}$^33^, so some works use a successive frame formula^9,21^ or discard frames with too few or too many pixels with counting^30^ to reduce it, while the SNR is reduced^8^. In PCB-SHWS, we only need the peak positions in the centroid distribution, rather than actual values which are influenced by the background, so we use the original covariance formula to calculate the JPD. Then, the centroid distribution is calculated from the JPD, and the peak positions are extracted using an algorithm and converted to gradient values. See Supplementary Information 1 for details of data processing, including the algorithms and a discussion about the SNR and the number of frames.

### Phase reconstruction

The two-dimensional Legendre polynomials^38^ ${L}_{m,n}({\boldsymbol{\rho }})={L}_{m}(x){L}_{n}(y)$, where

8 $${L}_{l}(x)=\frac{1}{{2}^{l}}\mathop{\sum }\limits_{k=0}^{\lfloor l/2\rfloor }\frac{{(-1)}^{k}(2l-2k)!}{k!(l-k)!(l-2k)!}{x}^{l-2k}$$

is defined on $-1\le x\le 1$. The gradient values are multiplied by 1.05 mm to rescale the ROI from 2.1 × 2.1 mm (7 × 7 apertures) to 2 × 2 with the dimension removed. We choose the maximum $m$ and $n$ to be 5 (higher modes are rapidly oscillating), so there are 35 modes excluding the constant phase mode ${L}_{0,0}=1$. For an aperture centered at $({x}_{0},{y}_{0})$ after rescaling, denoting ${L}_{l}{|}_{a}^{b}={L}_{l}(b)-{L}_{l}(a)$ and ${\varLambda }_{l}{|}_{a}^{b}={\int }_{a}^{b}dx{L}_{l}(x)$, the equations are

9 $$\begin{array}{l}{\kappa }_{x}=\frac{1}{4{a}^{2}}\sum _{m,n}{\alpha }_{m,n}{{L}_{m}|}_{{x}_{0}-a}^{{x}_{0}+a}{{\Lambda }_{n}|}_{{y}_{0}-a}^{{y}_{0}+a}\\ {\kappa }_{y}=\frac{1}{4{a}^{2}}\sum _{m,n}{\alpha }_{m,n}{{\Lambda }_{m}|}_{{x}_{0}-a}^{{x}_{0}+a}{{L}_{n}|}_{{y}_{0}-a}^{{y}_{0}+a}\end{array}$$

where $2a$ is the aperture width after rescaling ($2/7$ in our experiment). Considering all 49 apertures, we have 98 equations. The coefficients ${\alpha }_{m,n}$ are solved by the least square method, and the phase is reconstructed $\Phi ({\boldsymbol{\rho }})={\sum }_{m,n}{\alpha }_{m,n}{L}_{m,n}({\boldsymbol{\rho }})$.

## Supplementary information

Supplementary information for Position-correlated biphoton wavefront sensing for quantum adaptive imaging

## Data Availability

All data needed to evaluate the conclusions in this paper are present in the paper and/or the Supplementary Information. Additional data, including the raw camera frames, is available from the corresponding author upon reasonable request.
