# Supplementary material for: Position-correlated biphoton wavefront sensing for quantum adaptive imaging
Source: Light Sci Appl. 2025 Sep 8;14:311. doi: 10.1038/s41377-025-02024-4 (PMC12420783; doi:10.1038/s41377-025-02024-4)
Supplement: Supplementary file 1 — Supplementary information for Position-correlated biphoton wavefront sensing for quantum adaptive imaging [file 41377_2025_2024_MOESM1_ESM.pdf]

Supplementary information for
Position-correlated biphoton wavefront sensing for quantum adaptive imaging

Yi Zheng, Zhao-Di Liu, Jian-Shun Tang, Jin-Shi Xu, Chuan-Feng Li, and Guang-Can Guo

1. Details of data processing

When calculating the centroid distribution, if joint probabilities of all pixel pairs with the same centroid position are summed (even if they are far from each other and thus cannot have signals), the noise increases, so we choose to do the truncation: for two pixels (x_1, y_1) and (x_2, y_2), if $|x_1 - x_2|$ or $|y_1 - y_2|$ is greater than 300 µm, they do not contribute to the summing.

Before finding the peak positions, the background in the centroid distribution (due to the EMCCD count rate drift) is removed by this method: the value of each pixel is subtracted by the median of at most 9×9 pixels centered by this pixel. Figure S1 shows the distribution to be used and the cases without truncation or background removal.

In our phase measurement experiment, N ranges from 2.8×10^5 to 3.6×10^5. Figure S2 shows the centroid distributions (with truncation and background removal) from the no-phase data using only 2×10^4 and 4×10^4 frames. From the centroid distributions inside the center aperture from various numbers of frames N ranging from 5×10^3 to 2.8×10^5, we calculate their SNRs. The signal value is taken to be the average value of the 2×2-pixel region inside the center aperture with the

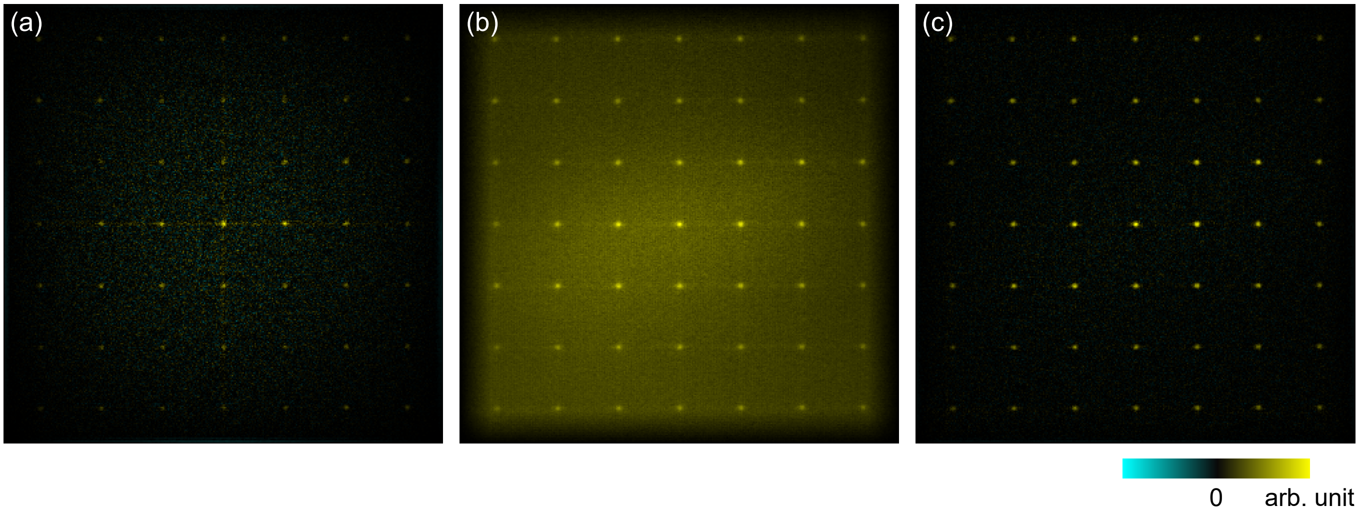

FIG. S1. The calculated centroid marginal distribution from the no-phase data (a) without truncation, with background removal; (b) with truncation, without background removal; (c) with truncation and background removal, which is to be used to extract the gradients.

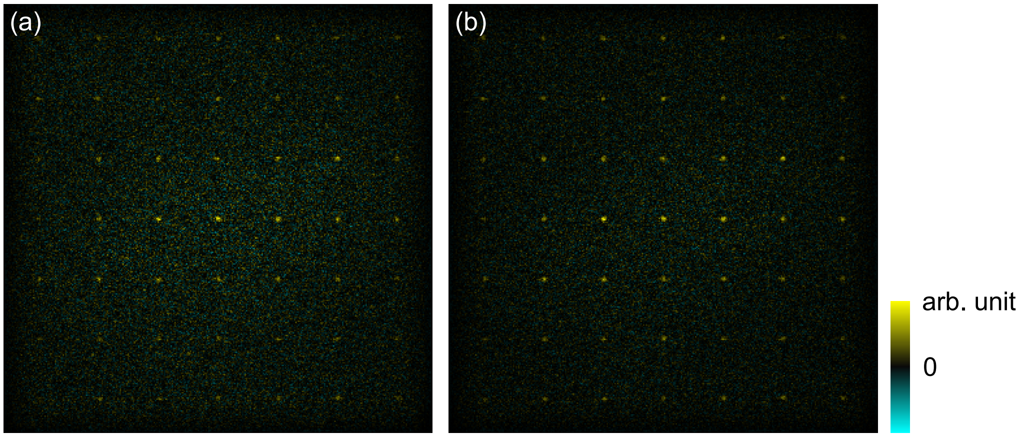

FIG. S2. The calculated centroid marginal distribution from the no-phase data with truncation using (a) 2×10^4 frames; (b) 4×10^4 frames.

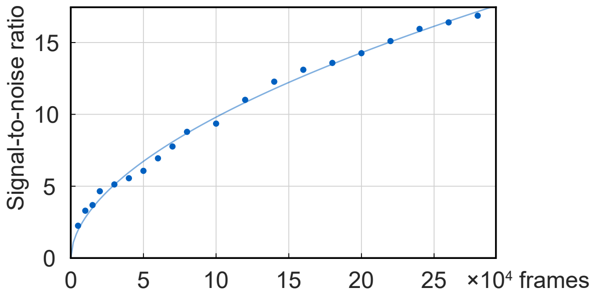

FIG. S3. The relation of the signal-to-noise ratio and number of frames.

largest value sum, the noise value is the standard deviation of the values in the center aperture excluding the 10×10-pixel region centered by the aforementioned 2×2-pixel region, and the SNRs are calculated and fitted by the power function $0.01888N^{0.5431}$ ($R^2 \approx 0.9944$), as shown in Fig. S3. So, the experiment could be faster if one would like to quickly estimate the phase. The frame rate of the EMCCD with the ROI set to 165×165 pixels is about 37 frames per second, so it only takes about 20 minutes for the phase to be properly measured.

In each aperture of the centroid distribution after background removal, the peak position is calculated by first finding a 4×4-pixel (with half the EMCCD pixel width 6.5 μm) region with the largest value sum and then calculating the average position in the 8×8-pixel region centered by the 4×4-pixel region weighted by the value of the distribution, so that the influence of areas without the peak is reduced. Under the paraxial approximation, the phase gradient of the aperture $\boldsymbol{\kappa}$ is the average position relative to the aperture center multiplied by k/f_{SH}.

2. Data of the phase measurement experiment

Figure S4 shows the direct images of the five cases in the phase measurement experiment. By comparing the no-phase case with the other images, displacements of the faint object patterns (corresponding to the phase gradient) can be observed. Certainly, this method has a lower accuracy and cannot succeed when the object is not present.

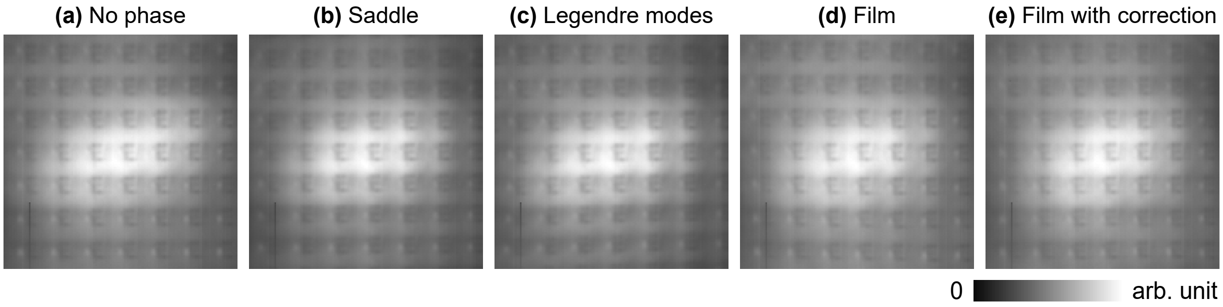

FIG. S4. The direct images by summing all the thresholded frames of the five cases.

Figure S5 shows the centroid distributions of the five cases with background removal and the peak positions identified by our algorithm, together with the exposure times and numbers of frames. The gradient distributions are shown in Fig. S6. The calculated Legendre coefficients (rounded to three decimal places) are shown in Table S1–S5 in the last page.

3. Experimental setup and principle of adaptive imaging

The setup of our adaptive imaging experiment is shown in Fig. S7.

To illustrate the impact of biphoton aberration on imaging based on the setup, for simplicity, we ignore the focal length differences or finite sizes of the Fourier lenses, let the pump beam be a plane wave, and use the momentum wavefunction to describe the biphoton field after an odd number of lenses. Letting the position wavefunction at the nonlinear crystal be $\psi_0(\boldsymbol{\rho}_1, \boldsymbol{\rho}_2) = c(\boldsymbol{\rho}_1 - \boldsymbol{\rho}_2)$, at the back focal plane of the first Fourier lens where the object $T(\mathbf{q})$ is placed, the wavefunction

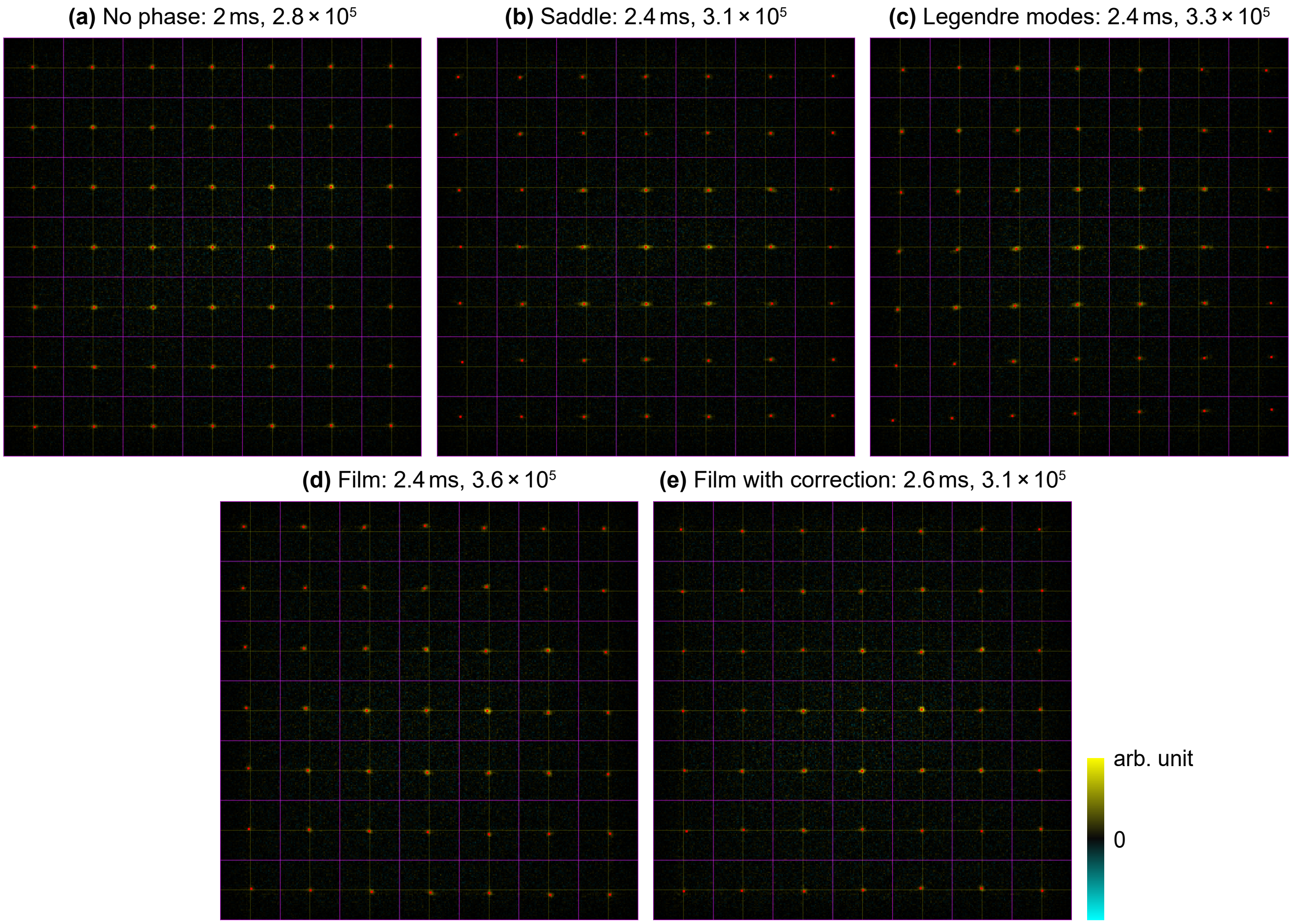

FIG. S5. The centroid distributions. Purple grids are aperture borders, yellow grid points are aperture centers, and red dots are the peaks identified by our algorithm. The EMCCD exposure time and number of frames taken are given for each case.

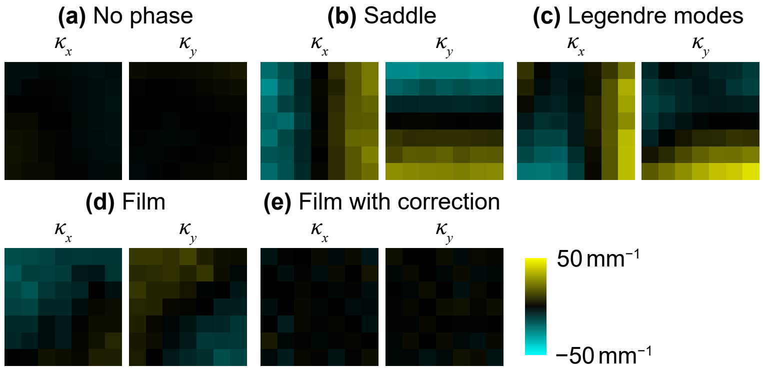

FIG. S6. The gradient (κ_x, κ_y) distributions. The no-phase case is the reference for all other cases.

becomes $\tilde{\psi}_1(\mathbf{q}_1, \mathbf{q}_2) = \tilde{c}(\mathbf{q}_1)T(\mathbf{q}_1)T(-\mathbf{q}_1)\delta(\mathbf{q}_1 + \mathbf{q}_2)$, where $\tilde{c}(\mathbf{q})$ is the Fourier transform of $c(\boldsymbol{\rho})$ [for collinear type-I SPDC, it has the form $\mathrm{sinc}(a|\mathbf{q}|^2)$, so we let $c(\boldsymbol{\rho})$ and $\tilde{c}(\mathbf{q})$ be even functions]. Denoting the effective image amplitude as $\tilde{c}'(\mathbf{q}) = \tilde{c}(\mathbf{q})T(\mathbf{q})T(-\mathbf{q})$ (also even), then $\tilde{\psi}_1(\mathbf{q}_1, \mathbf{q}_2) = \tilde{c}'(\mathbf{q}_1)\delta(\mathbf{q}_1 + \mathbf{q}_2)$. If the aberration source is regarded as a thin object, letting its amplitude transmittance be $T_a(\boldsymbol{\rho})$ whose Fourier transform is $h(\mathbf{q})$, the wavefunction at the imaging plane

$$\tilde{\psi}_3(\mathbf{q}_1, \mathbf{q}_2) = [\tilde{c}'(\mathbf{q}_1)\delta(\mathbf{q}_1 + \mathbf{q}_2)] * [h(\mathbf{q}_1)h(\mathbf{q}_2)]. \tag{S1}$$

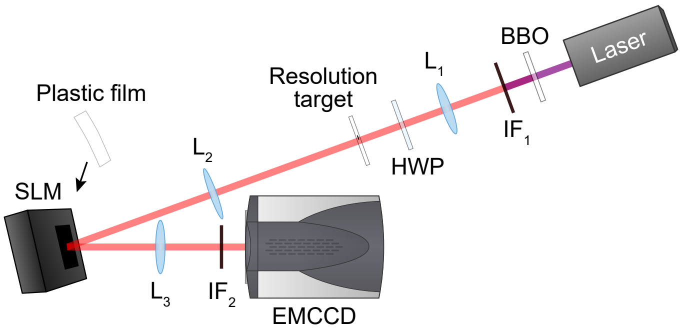

FIG. S7. The setup of the adaptive imaging experiment. The EMCCD sensor is at the back focal plane of L_3.

With no amplitude or phase modulation, $T_a(\boldsymbol{\rho}) = 1$, $h(\mathbf{q}) = \delta(\mathbf{q})$, and $\tilde{\psi}_3(\mathbf{q}_1, \mathbf{q}_2) = \tilde{\psi}_1(\mathbf{q}_1, \mathbf{q}_2)$, which means the effective image $|\tilde{c}'(\mathbf{q})|^2$ can be precisely obtained in both the direct image and the JPD of anti-correlated position pairs $|\tilde{\psi}_3(\mathbf{q}, -\mathbf{q})|^2$. If $T_a(\boldsymbol{\rho})$ is limited by an aperture, $h(\mathbf{q})$ and the centroid distribution have finite widths and the measured image is slightly blurry. Detecting the JPD of anti-correlated position pairs can realize quantum superresolution imaging at the standard quantum limit[1].

With a phase aberration $\arg T_a(\boldsymbol{\rho})$, $h(\mathbf{q})$ is generally wide. The wavefunction of anti-correlated positions

$$\tilde{\psi}_3(\mathbf{q}, -\mathbf{q}) = \int d\mathbf{q}_1' d\mathbf{q}_2' \tilde{c}'(\mathbf{q}_1') \delta(\mathbf{q}_1' + \mathbf{q}_2') h(\mathbf{q} - \mathbf{q}_1') h(-\mathbf{q} - \mathbf{q}_2') = \int d\mathbf{q}' \tilde{c}'(\mathbf{q}') h(\mathbf{q} - \mathbf{q}') h(-\mathbf{q} + \mathbf{q}') = \tilde{c}'(\mathbf{q}) * h_2(\mathbf{q}), \quad \text{(S2)}$$

where $h_2(\mathbf{q}) = h(\mathbf{q})h(-\mathbf{q})$ is also wide. So, the JPD of anti-correlated positions $|\tilde{\psi}_3(\mathbf{q}, -\mathbf{q})|^2$ is blurry and the object cannot be observed.

As for the direct image, one can analyze it classically. At the object plane, the light field with the intensity $|\tilde{c}(\mathbf{q})T(\mathbf{q})|^2$ is spatially incoherent in the first order. So, at the imaging plane, the intensity is $|\tilde{c}(\mathbf{q})T(\mathbf{q})|^2 * |h(\mathbf{q})|^2$, also vague.

4. Theory of phase measurement of anti-correlated biphotons

We consider photon pairs perfectly anti-correlated in position and with an infinite size $\int d\boldsymbol{\rho} |\boldsymbol{\rho}\rangle| - \boldsymbol{\rho}\rangle$. The anti-correlation center is unlikely to be the center, a side midpoint, or a vertex of an aperture, and thus for photons within a certain aperture, their entangled partners generally do not lie inside a single aperture, but at most 4 apertures. So, the microlens array should be laterally displaced to satisfy the requirement. However, effective methods to judge whether it is displaced correctly needs to be discovered before an actual experiment.

If a phase object $\Phi(\boldsymbol{\rho})$ is added to the photon pairs, the phase term of the resulting state

$$\int d\boldsymbol{\rho} e^{i[\Phi(\boldsymbol{\rho}) + \Phi(-\boldsymbol{\rho})]} |\boldsymbol{\rho}\rangle| - \boldsymbol{\rho}\rangle \quad \text{(S3)}$$

is an even function which we denote by $\Phi_{\text{eff}}(\boldsymbol{\rho}) = \Phi(\boldsymbol{\rho}) + \Phi(-\boldsymbol{\rho})$, which cancels if $\Phi(\boldsymbol{\rho})$ is an odd function up to a constant $\Phi(-\boldsymbol{\rho}) = C - \Phi(\boldsymbol{\rho})$. We consider photon 1 in an aperture with the width $2a$ centered by $\boldsymbol{\rho}_0$, and photon 2 in the opposite aperture centered by $-\boldsymbol{\rho}_0$ (temporarily masking other apertures). For Fourier transform, we displace the coordinate origin of photon 1 by $\boldsymbol{\rho}_0$, and photon 2 by $-\boldsymbol{\rho}_0$. Denoting the square region with the width $2a$ centered by $\mathbf{0}$ (the origin of the coordinate system for photon 1 or 2) as S, the state becomes $\int_S d\boldsymbol{\rho} e^{i\Phi_{\text{eff}}(\boldsymbol{\rho}+\boldsymbol{\rho}_0)} |\boldsymbol{\rho}\rangle| - \boldsymbol{\rho}\rangle$. Denoting $U(\boldsymbol{\rho}) = e^{i\Phi_{\text{eff}}(\boldsymbol{\rho}+\boldsymbol{\rho}_0)} \operatorname{rect}[\boldsymbol{\rho}/(2a)]$ and $\tilde{U}(\mathbf{q}) = \int d\boldsymbol{\rho} U(\boldsymbol{\rho}) e^{-i\mathbf{q}\cdot\boldsymbol{\rho}}$, the momentum wavefunction $\tilde{\psi}(\mathbf{q}_1, \mathbf{q}_2) = \tilde{U}(\mathbf{q}_1 - \mathbf{q}_2)$. When $\boldsymbol{\rho}$ is inside S, considering the spatial resolution requirement of SHWS, approximate $\Phi_{\text{eff}}(\boldsymbol{\rho} + \boldsymbol{\rho}_0)$ by $2\mathbf{q}_0 \cdot \boldsymbol{\rho}$ (ignoring the constant phase), and then $\tilde{\psi}(\mathbf{q}_1, \mathbf{q}_2) = \operatorname{sinc}_2[a(\mathbf{q}_1 - \mathbf{q}_2 - 2\mathbf{q}_0)]$, where $\operatorname{sinc}_2(\mathbf{q}) = \operatorname{sinc}(q_x)\operatorname{sinc}(q_y)$. If the two aperture are the same at the center ($\boldsymbol{\rho}_0 = \mathbf{0}$), $\mathbf{q}_0$ must be $\mathbf{0}$ from the even nature of $\Phi_{\text{eff}}(\boldsymbol{\rho})$. If not, by measuring the biphoton position difference $f_{\text{SH}}(\mathbf{q}_1 - \mathbf{q}_2)/k$ marginal distribution at the microlens focal plane, $\mathbf{q}_0$ can be determined from the difference between the peak position and the displacement from the photon 2 aperture to the photon 1 aperture. When the dynamic range requirement of SHWS is satisfied, there is no crosstalk from different pairs of apertures opposite to each other, so the phase gradient distribution, which should be an odd function, can be measured from the whole position difference distribution.

5. Difference between PCB-SHWS and quantum SHWS

In our previous work, the method named quantum SHWS[2] (we suggest another name "SHWS of biphoton joint phase" for it, since PCB-SHWS also involves the quantum optical field) was designed to reconstruct the phase of the four-dimensional biphoton spatial wavefunction $\psi(\boldsymbol{\rho}_1, \boldsymbol{\rho}_2)$. Its basic data processing methods are as follows. Letting the "aperture CPD" of one aperture be the sum of the CPDs (from the measured JPD at the back focal plane) with the other photon postselected to all pixels inside this aperture, it is used to extract the phase gradient distribution of the conditional wavefunction with the other photon postselected to this aperture which is approximated by a point. The dynamic range requirement of quantum SHWS is that photons passing through one aperture at the microlens must not escape to other apertures at the back focal plane, otherwise the calculated aperture CPD contains the contributions of the cases where the postselected photon is from other apertures. This can be judged from the camera direct image. If the larger spots (the biphoton field usually has a low spatial coherence in the first order) is still confined by their own apertures, the dynamic range requirement is satisfied, while Fig. S4 is a counterexample. After obtaining the phase gradient distributions of all apertures in the ROI, the four-dimensional phase can be reconstructed. Because the aperture needs approximation, the JPD and the phase gradient distribution of the biphoton state to be measured (right before the microlens array) should not vary rapidly at the scale of the microlens width, which is the spatial resolution limit of quantum SHWS. So, it only suits for biphoton states with much wider CPDs at the microlens array (e.g., with a weak or no position correlation or anti-correlation). Apart from the dynamic range and the spatial resolution, there are no other assumptions on the wavefunction. On the contrary, the theory of PCB-SHWS has assumed the biphoton has a strong position correlation, so the wavefunction of a biphoton state suitable for PCB-SHWS cannot be measured by quantum SHWS.

Since the position correlation (or the CPD width) of actual biphoton states must be finite, after magnifying the optical field many times using $4f$ systems, the resulting state can satisfy the spatial resolution requirement (the CPD width should be at least twice the microlens width) and seemingly applies to quantum SHWS. However, the whole beam is too large to be taken by a camera, and, most importantly, the much reduced phase gradients after magnification are imperceptible. So, if the biphoton state is known to have a strong position correlation, with an unknown phase added, the use of quantum SHWS is impractical.

The basic data processing method of PCB-SHWS is to calculate the biphoton centroid marginal distribution (which is two-dimensional) from the measured four-dimensional JPD. As the form of the biphoton state has been assumed, from the theory in the main text, the centroid distribution is an array of sharp peaks, which are then processed in the same way as classical SHWS to reconstruct the two-dimensional phase $\Phi(\boldsymbol{\rho})$. The dynamic range and the spatial resolution limit the form of $\Phi(\boldsymbol{\rho})$, rather than the four-dimensional wavefunction. A photon pair whose state is suitable for quantum SHWS are likely to go through different microlenses, so their centroid distribution at the back focal plane generally has no special properties.

Since the preparation of an arbitrary biphoton spatial state is difficult, we used quantum SHWS in a proof-of-principle experiment to reconstruct the biphoton wavefunction from SPDC after free-space propagation, where the weakening of the position correlation and the emergence of phase correlation had been predicted theoretically[3], while real applications of quantum SHWS rely on more mature multiphoton nonlinear modulation techniques or the realization of linear optical systems with desired impulse response functions[4]. Also, in our quantum SHWS experiment, millions of frames were taken for a desirable SNR, which took several hours for each biphoton state even using the faster Andor iXon Ultra 897 camera. For PCB-SHWS, the two-dimensional phase modulation of position-correlated biphotons is easy (by using an SLM), and the cancellation of an existing phase aberration can be checked by imaging[5]. Since the centroid distribution is used, taking tens of thousands of frames is enough. So, PCB-SHWS can be more easily applied in current quantum imaging techniques.

References

1. Giovannetti, V., Lloyd, S., Maccone, L. & Shapiro, J. H. Sub-Rayleigh-diffraction-bound quantum imaging. *Phys. Rev. A* **79**, 013827 (2009).

2. Zheng, Y. et al. Characterizing biphoton spatial wave function dynamics with quantum wavefront sensing. *Phys. Rev. Lett.* **133**, 033602 (2024).

3. Chan, K. W., Torres, J. P. & Eberly, J. H. Transverse entanglement migration in Hilbert space. *Phys. Rev. A* **75**, 050101(R) (2007).

4. Zheng, Y., Xu, J.-S., Li, C.-F. & Guo, G.-C. Theory of the monochromatic advanced-wave picture and applications in biphoton optics. *Phys. Rev. A* **110**, 063710 (2024).

5. Cameron, P. et al. Adaptive optical imaging with entangled photons, *Science* **383**, 1142 (2024).

TABLE S1. Legendre coefficients of the no-phase case

m	0	1	2	3	4	5
$n=0$	N/A	−0.790	−0.972	−0.079	0.096	0.032
$n=1$	0.158	−0.172	0.776	−0.424	−0.027	−0.041
$n=2$	0.425	−0.124	0.396	−0.142	0.100	0.139
$n=3$	0.356	0.489	−0.178	0.064	−0.013	0.013
$n=4$	0.004	0.006	0.039	0.100	−0.009	0.053
$n=5$	0.001	−0.042	0.030	−0.003	−0.054	−0.011

TABLE S2. Legendre coefficients of the saddle phase case

m	0	1	2	3	4	5
$n=0$	N/A	0.450	9.787	0.031	−0.538	−0.065
$n=1$	0.438	−0.167	0.053	−0.087	0.272	−0.119
$n=2$	−10.443	0.093	−0.124	0.127	0.412	0.201
$n=3$	−0.249	0.385	−0.138	0.595	−0.164	0.146
$n=4$	−0.174	−0.044	−0.282	−0.206	−0.547	−0.174
$n=5$	0.064	−0.312	0.185	−0.094	−0.170	0.557

TABLE S3. Legendre coefficients of the Legendre modes case

m	0	1	2	3	4	5
$n=0$	N/A	−0.655	8.034	3.748	−0.296	−0.039
$n=1$	0.881	6.203	−5.111	0.099	−0.026	−0.006
$n=2$	−7.633	−3.508	−0.142	0.412	−0.004	−0.076
$n=3$	3.102	−0.220	−0.129	−0.115	−0.144	0.017
$n=4$	−0.061	−0.206	−0.248	−0.278	−0.651	−0.809
$n=5$	−0.041	0.097	0.280	0.283	−0.442	−0.187

TABLE S4. Legendre coefficients of the film case

m	0	1	2	3	4	5
$n=0$	N/A	−5.606	2.581	0.089	−0.247	−0.164
$n=1$	0.603	−8.585	0.003	−0.301	−0.288	−0.043
$n=2$	3.321	1.312	−2.029	−0.343	0.345	0.202
$n=3$	0.122	0.174	−0.342	0.260	0.563	0.391
$n=4$	−0.204	0.017	0.349	0.327	−0.044	0.300
$n=5$	−0.047	−0.179	0.397	0.242	0.475	0.595

TABLE S5. Legendre coefficients of the film with correction case

m	0	1	2	3	4	5
$n=0$	N/A	−0.329	0.198	−0.227	−0.077	0.111
$n=1$	0.368	0.320	−0.252	0.001	0.123	−0.450
$n=2$	0.022	−0.046	0.322	0.136	0.243	−0.168
$n=3$	−0.282	−0.319	−0.101	−0.314	−0.585	−0.190
$n=4$	0.160	−0.178	0.141	−0.145	0.484	−1.109
$n=5$	0.179	0.265	−0.039	−0.546	−0.075	−0.005
